# Hospital and Institutional News

**Published:** 1918-05-25

**Authors:** 


### HOSPITAL AND INSTITUTIONAL NEWS.

### THE AFTER-CARE OF DISCHARGED WARRIORS.

On Whit Monday the Duke of Connaught opened the Inter-Allied Conference on the after-care of discharged sailors and soldiers at the Central Hall, Westminster. The gathering of representatives from Belgium, France, Italy, Portugal, Serbia, Siam, Greece, and the United States of America, besides those from the Dominions and India, was in itself an interesting sight, and one that promised well for practical results from the daily discussions and visits to English centres of effort. The Duke read his speech in French, and those in reply were in the same language. Later the King and Queen with Princess Mary visited the exhibition of work by disabled men. It is to be hoped that the public will largely follow their example, as the work exhibited is of great interest, and comprises specimens from other countries as well as from a number of different workshops and training centres in our own country. Men from St. Dunstan's and the Cordwainers' College may be seen at work mending boots, and upholstery is being done by disabled men in another place. Probably to many the variety of work done by disabled men will come as a surprise, the Italian bookbinding and tooling, with chair-seats and other work in leather, and the parasol and cane work are very remarkable. Serbians and some of our men, especially in the Lord Roberts' Workshops, have turned the metal and wood of shells and captured aeroplanes to good account. Even lay people can learn a good deal from the exhibits and photographs of surgical repairs to damaged human frames. On Monday afternoon the cinema was put to good purpose to illustrate curative surgery by massage, gymnastics, and mechanical exercises.

### SECTIONAL SUBSCRIPTIONS IN LONDON.

The difficulty of defining the district which a London hospital serves is well seen in the case of the Bolingbroke Hospital. It receives patients and accident cases from Balham, Tooting, Wandsworth, Battersea, and Earlsfield. There is no uncertainty about that. But when its list of subscribers is searched we find that it relies on the great funds and a mere handful of annual subscribers, and that the income from these together bears no sort of relation to the half-million people in this area. A London district is not a unit; it has no common bond except its geography, and that is artificially drawn and unrecognised by its inhabitants. The consequence is that a small London hospital does not know where to look for support, and has no financial policy except a series of sporadic appeals when its overdraft becomes intolerable. The Bolingbroke Hospital, however, bases its latest appeal on the claim that it does much service to "the industrial classes by whom it is surrounded." The latter words suggest that its committee should not be satisfied with an appeal, but should attempt to organise a weekly collection in the neighbouring works or shops. Many people even so would escape their obligation because their home and not their factory is in the district. But there should be enough to save the situation, and we cannot see why weekly collections should not be started in any works in a district which sends patients to the hospital. Care would be necessary to avoid competition with the Saturday Fund, but the plan should be adopted as far as possible.

### A GUIDE THROUGH THE OFFICIAL MAZE.

One of the drawbacks of treating military patients in civil hospitals has always been the necessity for filling up innumerable forms and of the hospitals adapting themselves, as far as possible, to the requirements of the official machine. This is not always an easy task, and when the morning's post brings its regular quota of circulars in imitation typewriting, half-a-dozen in each envelope, dealing with a variety of subjects, from the payment of medical officers to the establishment of a local Pensions Committee at Llanuwchllyn, the secretary may well give an exclamation of impatience as he sorts the red-tape from the chaff. Probably the Government Departments concerned are no less worried by the difficulty of bringing liberty-loving institutions into official harness. Fortunately, most hospitals have the advantage of being affiliated to a central military hospital, which extends to them a protecting wing. Thus recently when many hospitals were staggered by the receipt of a parcel of "Temporary Allowance Books" from the Post Office, without explanation, the central hospital soon stepped in and relieved them of the incumbrance—also without explanation. Specially to be commended in this regard is the Queen Alexandra Military Hospital, Millbank, whose registrar a short time ago gave a lecture explaining in detail the working of the official machinery in regard to military patients. To this lecture the secretaries and clerks of hospitals affiliated to Millbank were specially invited, to the advantage of all concerned.

### "SACRILEGE" AT A MILITARY HOSPITAL.

Southwark Board of Guardians is making a determined stand against an attempt at short notice to convert the chapel at the Southwark Military Hospital, formerly the infirmary, into a recreation room for wounded soldiers. The Visiting Committee report that as there are over one hundred vacant beds in the hospital there is no urgency to justify the conversion. The board has instructed the clerk to enter the strongest protest to the War Office against the step proposed, and to communicate with the Bishop of Southwark and the chaplain on the subject. Some better place must be found. In summer there is less need for an inside recreation-room, especially at the Southwark Military Hospital, which is surrounded by lovely grounds and gardens.

### THE BRADFORD SCHOOL CLINIC CRITICISED.

Mr. Herbert Gill, in presiding over the recent quarterly meeting of the Bradford Hospital Fund, alluded to the abuse of the Royal Infirmary by the municipal school clinic. He said that the clinic had sent fifty-seven cases to the hospital's out-patient department, and ten in-patients, although the clinic possessed a staff of doctors and nurses paid by the City Council to do this work. It is neither right nor fair, he added, and the ratepayers should know. Apparently the Royal Infirmary is not the only hospital to receive cases from the clinic; the Royal Eye and Ear Hospital has also had a share of them. What is the explanation? What is the staff of the clinic doing?

### THE NEW CALL-UP AND SUBSCRIPTIONS.

In congratulating the workers of Keighley on the increase in the income of their fund for the benefit of Keighley Victoria Hospital, Mr. Charles Kitchen, the organising secretary, pointed out future difficulties. The calling up of so many men under the new Act would seriously reduce the number of contributors. Since the industries from which many men are to be drawn will be maintained at full vigour, while an increase in the number of women workers is to be expected, why does Mr. Kitchen foresee a diminution in the number of contributors? Has past experience indicated this? Is the new Act a new revolution?

### THE ULTIMATE TEST OF AN APPEAL.

How far a special appeal, particularly in London, gains permanent new friends to a hospital perhaps depends upon the amount of care subsequently taken to preserve them. The Mayor of Hampstead lately declared that the special appeal which was issued in March last year had accomplished a splendid work in getting new subscribers to Hampstead General Hospital, and this seems to show that permanent new friends have been gained. Mr. Harold Wigg, the secretary, is not the kind of administrator to allow their regard to wither for want of attention, and though the appeal was started three months before his appointment, we have no doubt that its promise of permanent success already owes much to his conscientious work. It is work of this character which alone is fruitful in the long run, and a hospital is to be envied which possesses a secretary of whom, as in the present case, conscientiousness is a proved quality. The brilliant men are mostly those who leave to others the maintenance of the ideas which they invent. But in hospital work, as in other walks of life, the race is not to the swift, but to the persevering. The tortoise may be outshone at the start by the hare, but the tortoise carries home and keeps the best prizes.

### A "STRIKE" OF COLLIERY DOCTORS.

Mr. T. H. Cann, secretary of the Miners' Association, lately criticised the colliery doctors for "striking" for an increased fee, and described their action as one of refined cruelty. In certain districts, he said, the doctors had wholly refused to attend anyone, even in cases of serious illness. Further, he declared that some of them had declined to attend the wives of the miners during their confinement unless the husbands signed an agreement that they would give the advanced fee demanded. Mr. Cann, however, added that a sub-committee of his Association and the representatives of the British Medical Association had received an assurance from the latter that the Association would endeavour to persuade the doctors "who went on strike" to treat maternity cases as private patients. This is another instance of the evils which attend club practice and panel work.

### A HOSPITAL SECRETARY'S RETIREMENT.

Mr. F. G. Steed has resigned the post of honorary secretary to St. Leonard's Hospital, Sudbury. After twelve years of continuous work his health has broken down, and led to his resignation. Mr. Steed has the satisfaction of leaving the hospital at the close of a financial year with no current deficit, and at a time when three gifts have already been received in memory of those fallen in the war. A local memorial to these men is under discussion, and there is reason to hope that it may take the form of giving the hospital additional beds and a children's ward. If these additions were made an east wing the building would become uniform. Mr. Steed retires, then, on the eve of future developments, and this in itself is a tribute to his work.

### HOSPITAL PLANS IN ACTON.

Mr. E. Monson has submitted plans of a new ward for ten beds, two private wards, and three additional nurses' bedrooms to the Acton Hospital Council. The cost of the new wards is expected to be £1,500, and of the three new bedrooms £300. The District Council has requested that certain diseases, which the Local Government Board declares to come within the purview of the council, should be treated at the hospital. An alternative proposal is for the District Council itself to build a hospital. Mr. Monson's plans have been approved, however, and the hospital council has sent them to the three Funds and to the Local Government Board for their approval, criticism, and, in the case of the Funds, in the hope of a contribution. The District Council, it is understood, will pay the hospital for the patients treated a sum sufficient to meet the cost of maintenance.

### A WEAK DEFENCE OF THE LETTER SYSTEM.

In congratulating the Northamptonshire Hospital Week Fund on having reached a total of £5,341, the recent report of the Committee says: "Comparing the thirteen years which have elapsed since the adoption of the letter system with the thirteen years prior to the adoption of the system, which has been such a benefit to workers in factories, the former period showed an increase of £20,378 on the total amount paid to the hospital, £11,922 being the sum total for the thirteen years during which the letter system was not in force." This is clumsily expressed, and is it not hasty to assume that the increase in the later period has been due to the letter system rather than to the increase in the population and the organisation of hospital funds? The letter system is open to much objection, and cannot claim the rewards of time in this way.

### RHYL RED CROSS HOSPITAL.

The public have responded so well to the Rhyl Red Cross Hospital that at the end of the year the committee finds itself with £1,200 in hand. Since the hospital opened its doors, 672 men have passed through, besides the discharged soldiers who have been treated at the request of the Pensions Committee. Mr. C. J. Turner has presented the hospital with a billiard table, so that the treatment and recreation of the patients are alike objects of his generosity.

### STETHOSCOPE BOILS.

When we see the house-man walking from ward to ward with his stethoscope carelessly hanging round his neck, hardly one of us ever dreams that this simple method of carrying an implement of his craft is dangerous to the carrier. But Dr. Herbert French, in a clinical lecture delivered at Guy's Hospital, shows that he has more definite knowledge on the subject. He says that medical men and medical students are from time to time bothered by painful boils on either side of the neck, behind and a little lower than the ears. In a large proportion of cases these boils are due to nothing whatever but the stethoscope; they are a consequence of the habit of releasing the ear-pieces of the stethoscope from the ears and allowing them to catch on the neck, where they implant any organisms they may have picked up in the ear, or may rub the skin sufficiently to allow ingress of the local staphylococci. Dr. French has seen students suffer from these boils for months or years in spite of all kinds of treatment, and cease to suffer from them when the habit of carrying the stethoscope with its ear-pieces on the neck was discontinued.

### THE HEALTH OF GERMAN WOMEN.

The accounts which reach us of the conditions of food and comfort prevailing in Germany make it clear that the public health of that country is unsatisfactory. Diseases of malnutrition are frequent, and, especially among women, the ratio of illness is much greater than in normal times. Working women are not only "overdone," but are suffering from the scarcity, dearness, and poor quality of food and the trying conditions under which it has to be obtained. As a rule there is not much to choose, in point of physique, between the working women of one Western country and another; but in Germany the middle-class Teutonic woman has hitherto led a less active, open-air life than her English congener. Were we to suffer privations similar to those which afflict Germany our women might maintain their health more successfully than theirs.

### HOSPITALS AND A RUMOUR.

Mr. Macpherson in the House of Commons has declared, in answer to Mr. Gilbert, that reports obtained from various hospitals in the London district do not suggest to the medical authorities that any wounds to our soldiers have been caused by a new kind of explosive, chemical shell, or gas used by the enemy.

### LIP-READING FOR DEAF SAILORS AND SOLDIERS

The committee of the Royal School for the Deaf, Margate, has presented to the Ministry of Pensions, as a hostel for the period of the war, a beautifully situated residence in the school grounds, fully staffed, for domestic care and instruction. A minimum charge for food only will be made from the supplementary pension allowances paid to the men who are willing to participate.

### WORKHOUSES AFTER THE WAR.

Will the workhouses which have been commandeered by the Army Council for hospitals ever again be utilised as Poor-Law institutions? For instance, at the Brighton Workhouse, which is one of the largest of the kind in the south, enormous sums have been spent to bring the institution up to hospital requirements. Brighton Workhouse was, firstly, transformed to meet the requirements of the different castes of Indian soldiers, and secondly, on their withdrawal further large sums of money were spent to prepare it for British soldiers. As it at present exists, the institution is a great hospital catering principally for Colonials, and further adaptations may be required if the necessity arises for a great hospital for American soldiers within easy distance of France. After all these expenditures, even to talk of restoring the premises for Poor-Law purposes seems a difficult matter. And those workhouses which the Army Council has taken over will be needed for some time after the conclusion of peace, and every day brings us nearer to the Ministry of Health, whose functions will hardly perpetuate the unpopular Poor-Law system.

### SURGERY UNDER THE POOR LAW.

The Bethnal Green Board of Guardians has come to the conclusion that it would be in the interests of the patients if an assistant medical officer was appointed who was a skilful surgeon but who would assist with the ordinary medical work of the institution. The Board has accordingly appointed Dr. G. Cooke as assistant at a salary of £350 a year.

### TUBERCULOSIS MORE RIFE.

According to Dr. F. Barretto, acting tuberculosis officer, East Riding County Council, tuberculosis appears to be at the present time more rife and more fatal than it was previous to the war. The death-rate has increased in both sexes, from which it may be inferred that adverse conditions have arisen. He insists upon proper housing, adding that unless preventive measures are carried out no satisfactory result can be expected.

### THE NEW MATERNITY BILL.

The new Maternity and Infant Welfare Bill which enables local authorities to provide for the health of expectant mothers, nursing mothers, and children under the age of five, has been discussed by the Metropolitan Standing Joint Committee of

Borough Councils. Whilst agreeing with the general principle of the Bill, the Joint Committee was opposed to the provision which gives to the London County Council powers identical with those given to the Borough Council for making arrangements with regard to maternity and child welfare. The Borough Councils are the local authorities under the Notification of Births Act, to the expressed exclusion of the County Council; and so the Joint Committee decided to make representations to the Local Government Board accordingly. The opinion was expressed that the power to appoint as members of maternity and child welfare committees persons who are not members of the appointing Councils should be optional and not obligatory.

### A PROGRESSIVE DISEASE.

IT seems that scabies is going to cause trouble. A report upon the Hull school medical service says that last year there was an increase of 50 per cent. in the number of cases. The disease is obviously spreading progressively year by year, and if the present rate of increase is maintained it will soon be very difficult to avoid infection. This experience is not confined to Hull, but has been mentioned in L.C.C. reports and by many school medical officers in the provinces. Usually it is explained that the disease has been introduced by soldiers from the Front who have acquired it in the trenches. Paris also reports a large increase in the prevalence of the disease. As the Hull report says, the homes of many of the children are infected with the disease, and so are their relations not of school age. Children, therefore, are frequently reinfected.

### GOVERNMENT BLANKETS FOR INSTITUTIONS.

AT the Health Committee of the Southend Corporation the Medical Officer of Health reported that he had made further inquiries from the Contracts Department of the War Office in regard to the purchase of a further fifty blankets for the sanatorium. He had now heard that the Director of the Wool Textile Production Department was prepared to supply blankets for institutions, of a standard size, at a net price of 17s. 4½d. per blanket. The medical officer was instructed to purchase fifty of these.

### DISPENSERS' SALARIES.

THE committee of the Royal Portsmouth and Gosport Hospital has decided to raise the salary of the dispenser to £200 per annum. The salary previously was £175.

### THE PROGRAMME FOR "BABY WEEK."

MR. HAYES FISHER will open on July 2, at the Central Hall, Westminster, the conference which the National Association for the Prevention of Infant Mortality is organising in connection with National Baby Week. The conference will last two days, and the chairmen include Sir Arthur Newsholme, M.D., and Sir Francis Champneys, president of the Central Midwives Board. Antenatal factors in infant mortality, and institutional *versus* domiciliary treatment of the lying-in mother, will be discussed. Dr. Harold Scurfield, medical officer of health for Sheffield, has a topical subject in "Mothers' Pensions," and in the afternoons and evenings of the two days six lectures on infant care, intended primarily for elementary school teachers, will be given. There is also to be an educational exhibition for the instruction of health visitors and infant welfare workers.

### THE COST OF MUNICIPAL CRECHES.

As a form of municipal trading, there is no immediate financial profit to be made out of crèches, although they provide a felt want. Birmingham, which opened a municipal crèche a year ago, reports that 269 individual babies were taken there, the total number of attendances during the day being 10,503, and during the night 9,439. The parents pay for the infants at the rate of 5s. 6d. per week for one child for day attendances, and 8s. 6d. per week for day and night. The charge is reduced for two or more children. The Board of Education pays a grant of 7d. per attendance day or night; but these payments do not nearly meet the total cost, and it is estimated that for the year the crèche will cost the ratepayers £1,350. The work is not now to be extended, though once three or more crèches were proposed, and we regret to hear that the present crèche will be discontinued as soon as the existing abnormal conditions admit. So long as the muncipal crèche meets a felt want, it is worth all the money that can reasonably be spent upon it. Its aim is not, and cannot be, to make a profit. Nor, indeed, can we know whether it "pays" until against the figures of its cost are set the "savings" represented by an increase in the infant health of the city. It is because savings of this kind cannot easily be calculated by a commercial auditor that a commercial audit tells one nothing worth knowing about this or any other experiment in municipal statesmanship.

### THIS WEEK'S DRUG MARKET.

THE market has hardly recovered from the recent holiday; few transactions have taken place, and there are few price changes. In several instances prices have a rather lower tendency, but for the most part this is due to temporary causes, and there is at present no reason to expect that the general upward tendency has come to a stop. Salicylic acid is in good supply, and there are offers at lower rates; sodium salicylate also has a slightly lower tendency, but this is not so pronounced as in the case of the acid. The market for bromides is inactive at the moment; but the scarcity of ammonium bromide has not yet been relieved, and the high price is well maintained. Caffeine is offered at rather lower prices. Russian cantharides are dearer. Citric and tartaric acids continue in demand. Arrivals of ergot of rye are expected, but in the meantime the high value is maintained. Guaiacol carbonate is offered at rather lower rates. The demand for menthol is very slack. Phenacetin still has a lower price tendency, and the same may be said of phenazone. American saccharin still sells at high prices.

